# Metagenomic Investigation of Pathogenic RNA Viruses Causing Diarrhea in Sika Deer Fawns

**DOI:** 10.3390/v17060803

**Published:** 2025-05-31

**Authors:** Weiyang Wang, Qilin Wang, Runlai Cao, Yacong Li, Ziyu Liu, Zhuqing Xue, Xiaoxu Wang, Zhijie Liu

**Affiliations:** Key Laboratory of Special Animal Epidemic Disease, Ministry of Agriculture and Rural Affairs, Jilin Provincial International Cooperation Key Laboratory for Science and Technology Innovation of Special Animal and Plants, Institute of Special Animal and Plant Sciences, Chinese Academy of Agricultural Sciences, Changchun 130112, China; weiyangwang8779@163.com (W.W.); wangqilin@caas.cn (Q.W.); caorunlai@caas.cn (R.C.); lycong910@163.com (Y.L.); 13844790017@163.com (Z.L.); xzqblblbl@163.com (Z.X.)

**Keywords:** sika deer, viral metagenomics, whole-genome sequencing, rotavirus

## Abstract

Diarrhea is a common disease in sika deer. The causes of diarrhea in sika deer are complex and involve a variety of pathogens. Additionally, new virulent pathogens are continuously emerging, which poses a serious threat to deer’s health and particularly affects fawns’ survival rate. In the present study, feces samples were collected from fawns with diarrhea in Jilin Province, in the northeast of China. The viral communities were investigated using the metagenomic method. Viral metagenome data revealed that the viruses in the fecal samples were mainly from 21 families in 14 orders. The major viruses in high abundance were astrovirus, rotavirus, coronavirus, and bovine viral diarrhea virus. In addition, a large number of phages, which mainly belonged to the family *Siphoviridae*, were identified. Then, the known causative virus species were investigated via RT-qPCR. The results showed that the infection rates of bovine coronavirus, bovine rotavirus, and bovine viral diarrhea virus were 59.44%, 58.89%, and 21.67%, respectively, and mixed infections were commonly seen in the samples. A bovine rotavirus strain was successfully isolated from the positive samples. Whole-genome sequencing revealed that the genotype of the strain was G6-P[1]-I2-R2-C2-M2-A3-N2-T6-E2-H3, indicating the recombination of rotavirus. This study revealed the profiles and characteristics of viruses that cause sika deer diarrhea, which will be helpful for understanding diarrhea diseases in sika deer.

## 1. Introduction

Diarrhea is a prevalent condition characterized mostly by loose stool and stomach pain. Its prevalence is significantly high, and it is associated with a wide range of diseases, posing a considerable threat to human and animal health [1,2]. Globally, approximately half a million children die each year due to diarrhea [3]. Diarrhea not only leads to the significant loss of water and electrolytes, resulting in an acid–base imbalance [4], but it also may cause malnutrition, resulting in reduced production performance, such as weight loss and delayed growth and development of the affected animals [5,6]. Moreover, prolonged diarrhea can result in vitamin insufficiency, subsequently impacting patients’ clinical presentations. Diarrhea can significantly impair the body’s immune system, rendering patients more vulnerable to other pathogens and secondary illnesses, which can be life-threatening in severe instances [7].

In livestock, diarrhea is the cause of elevated mortality rates among young animals. While diarrhea can occur throughout the year, its incidence increases between fall and winter [8]. The causative diarrhea pathogens in young animals include bacteria, viruses, and parasites [9,10,11,12]. Viral infection is the primary cause of diarrhea in young animals. It is known that coronavirus, rotavirus, infectious gastroenteritis virus, and parvovirus are the leading causes of infection [13,14,15,16]. Using high-throughput sequencing technology approaches, novel viruses, including porcine deltacoronavirus and porcine kobuvirus, have recently been identified [17]. The emergence of these pathogens has increased the complexity of preventing and controlling viral diarrhea in young animals. Sika deer are distributed mainly in China, Japan, the Russian Far East, the Korean Peninsula, and Vietnam [18,19]. Diarrhea caused by bovine viral diarrhea virus (BVDV) has been shown to significantly restrict the sika deer farming industry because of its high morbidity and mortality [20,21,22]. In addition, many undetected viruses pose potential threats to the health of sika deer [12].

Viral metagenomics is an advanced tool for investigating viral populations in particular habitats [23]. This approach overcomes the low-throughput constraints of conventional detection methods and can identify multiple species in mixed samples. This approach is efficient and important for discovering novel pathogens and investigating genetic evolution [24]. Currently, this method has been extensively utilized in many fields, such as marine ecological studies [25], agronomy [26,27,28], human genetics, and diagnostics [29].

The overall profile of the pathogens that cause diarrhea in sika deer is unclear. This study used viral metagenomic technology to examine the RNA virus profile of diarrheal stool samples from sika deer in Jilin Province, China. The genetic features of the identified virus species were analyzed, and a *rotavirus* strain was isolated and characterized. The data obtained in the present study will be helpful for understanding and preventing diarrhea diseases in sika deer.

## 2. Materials and Methods

### 2.1. Ethical Approval

The samples were collected by professional veterinarians and evaluated by the Animal Infectious Disease Prevention and Control Team of the Institute of Special Animal and Plant Sciences, Chinese Academy of Agricultural Sciences.

The People’s Republic of China’s Regulations for the Administration of Affairs Concerning Experimental Animals were followed in treating the laboratory animals. All experiments were approved by the Ethics Committee of the Institute of Special Animal and Plant Sciences, Chinese Academy of Agricultural Sciences (Approval No. ISAPSAEC-2023-038SDF).

### 2.2. Collection of Fecal Samples and Processing

Samples were collected from June to September 2023 and 2024 in Shuangyang District, Changchun City and Dongfeng County, Liaoyuan city, Jilin Province, the main breeding areas of sika deer in China. A total of 360 fecal samples from fawns with diarrhea were collected. The animals were induced to defecate by anal sphincter stimulation, and each sample was collected directly into a 50 mL sterile centrifuge tube. One hundred fifty samples were randomly selected and divided into five groups (*n* = 30 per group). In each group, 1 g (±0.01 g) of each of the 30 individual samples was mixed to form 5 pooled samples (A1–A5). All the samples were stored at −80 °C for future use.

### 2.3. RNA Isolation

The pooled samples were diluted with 900 µL of sterile phosphate-buffered saline (PBS; pH 7.2) and homogenized using a vortex mixer for 2 min. After standing for 10 min, 300 µL of the supernatant was transferred to a 1.5 mL RNase-free centrifuge tube. Then, 900 µL of TRIzol reagent (Thermo Fisher, Waltham, MA, USA) was added, mixed by a vortex mixer, and incubated at room temperature for 10 min. Afterward, the mixture was centrifuged at 12,000 rpm for 5 min at 4 °C. The supernatant was transferred to a new 1.5 mL RNase-free tube, and 200 µL of chloroform (Sigma-Aldrich, St. Louis, MO, USA) was added. The mixture was inverted to mix, incubated at room temperature for 10 min, and centrifuged again at 12,000 rpm for 10 min at 4 °C. The upper layer was carefully pipetted into another 1.5 mL RNase-free tube, and 500 µL of isopropanol (Sigma-Aldrich, St. Louis, MO, USA) was added. After gentle inversion, the mixture was incubated at room temperature for 10–30 min and then centrifuged at 12,000 rpm for 10 min at 4 °C. A white pellet formed at the bottom of the tube, and the supernatant was discarded. The pellet was washed with 75% alcohol, followed by another centrifugation at 12,000 rpm for 10 min at 4 °C. The alcohol was discarded, and the pellet was air-dried in a laminar flow cabinet. Finally, the pellet was dissolved in 60 µL of RNase-free water. A mixture of 9 µL of total RNA and 1 µL of 10× loading buffer was run on a 1% agarose gel for 30 min of electrophoresis. The gel was then monitored under UV light, and only the samples whose 28S and 18S rRNA bands were visible were considered high quality. The qualified RNA samples were subjected to high-throughput sequencing at Novogene Co., Ltd. (Beijing, China).

### 2.4. Viral Metagenomic Library Construction and High-Throughput Sequencing

The TIANSeq rRNA Depletion Kit (TIANGEN, Beijing, China) was used to remove host and symbiotic microbial ribosomal RNA (rRNA) according to the manufacturer’s instructions. The remaining RNA was fragmented into 250–300 base pair (bp) fragments using divalent cations in an NEB fragmentation buffer. The fragments were used as templates for the synthesis of the first strand of cDNA with random oligonucleotide primers. A library was constructed using the Fast RNA-seq Lib Prep Kit V2 (ABclonal, Wuhan, China), followed by PCR amplification. A Qubit 2.0 fluorometer was used for preliminary quantification, and the library was diluted to 1.5 ng/µL accordingly. The insert size of the library was determined via an Agilent 2100 bioanalyzer. The concentration of the library was quantified by RT-qPCR. PE150 high-throughput sequencing was performed using the Illumina NovaSeq 6000 platform.

### 2.5. Bioinformatics Analysis

The bioinformatics analysis was performed according to a standard protocol developed by MAGIGENE, Guangzhou, China. The key procedures include quality control of the raw reads and removing low-quality data using Trimmomatic software [30]. Eventually, the assembled contigs’ nucleic acid and amino acid sequences were aligned, and the annotation results were analyzed. International Committee on Taxonomy of Viruses (ICTV) criteria (accessed on 22 May 2025, from https://ictv.global/report) were followed for Blast analysis of the putative new genus or species of viruses described in this study (Supplementary Table S1).

### 2.6. Real-Time PCR

BVDV, BCoV, and BRV were amplified using real-time PCR detection kits (TopGeneBio, Qingdao, China). Fifty microliters of the fecal sample diluent were added to a 200 μL clean centrifuge tube. Then, 100 µL of lysis buffer was added, and the mixture was vortexed thoroughly. Before the experiment, eight tubes preloaded with 10 µL of the premixed solution and the detection reagent were equilibrated to room temperature. After instantaneous centrifugation, 5 μL of detection reagent and 5 µL of sample cracking solution were added. The fluorescence signal was detected using a real-time fluorescence quantitative PCR instrument (Applied Biosystems StepOnePlus™ Real-Time PCR System, Thermo Fisher Scientific, Waltham, MA, USA).

### 2.7. Isolation and Identification of Rotavirus

BRV-positive fecal samples were diluted with sterile PBS and then centrifuged at 8500 rpm for 10 min at 4 °C. The supernatant was filtered through a 0.22 µm pore filter (Merck Millipore, Darmstadt, Germany), and trypsin (Sigma-Aldrich, St. Louis, MO, USA) was added to a final concentration of 20 µg/mL, followed by vortex mixing. The mixture was activated for 1 h at 37 °C in a 5% CO₂ incubator. The samples were then inoculated onto Vero cell monolayers in 25 cm^2^ culture flasks and incubated for 1 h at 37 °C. The suspension was subsequently discarded, and the virus maintenance medium supplemented with 5 µg/mL trypsin was added and incubated at 37 °C with 5% CO₂. The cells were blind passaged for 3 to 5 passages at 37 °C with 5% CO₂ [31]. Vero cells infected with *rotavirus* were used as the experimental group, and untreated Vero cells were used as the control group. The cells were fixed with 4% formaldehyde for 30 min, washed three times with PBS, and permeabilized with Triton X-100 (Solarbio, Beijing, China) for 10 min at room temperature. The cells were blocked with 5% bovine serum albumin (FBS, Invitrogen, Carlsbad, CA, USA) for 1 h. The cells were subsequently incubated with a 1:400 dilution of monoclonal antibody against *rotavirus* VP6 (Zoonogen^®^, Beijing, China) and a 1:10,000 dilution of FITC-conjugated goat anti-mouse IgG (Invitrogen, Carlsbad, CA, USA) at 37 °C for 1 h each. Finally, DAPI (5 μg/mL) (VectorLabs, Newark, CA, USA) was added to the samples, which were subsequently incubated in the dark for 10 min, after which the immunofluorescence staining was observed, and images were captured using a fluorescence microscope (Leica, Hessen, Germany). The infected Vero cells were collected by centrifugation at 3000 RPM for 30 min at 4 °C. The sediment was subsequently used to observe the morphology of the virus particles using transmission electron microscopy (TEM, Hitachi TME HT7800, Tokyo, Japan).

### 2.8. Whole-Genome Sequencing and Assembly of the Rotavirus Genome

The RNA samples extracted from infected Vero cells were sent to Guangdong Megger Gene Technology Co., Ltd. for whole-genome sequencing. The RNA samples were randomly disrupted for reverse transcription and two-strand synthesis to obtain cDNA. The obtained double-stranded cDNA was repaired by end ligation, and the base “A” was added to the 3′-end so that the cDNA fragment could be connected to the 3′ splice site with the base “T”. These DNA fragments with the adaptor were amplified by PCR to complete library construction. The libraries were subjected to sequencing [32].

After sequencing, SOAPnuke software (v.2.0.5) [33] was used to remove the spliced and low-quality paired reads. Clean reads were aligned to the host genome via SOAP aligner or BWA software (v.0.7.17) [34], and host sequences were removed. High-quality reads were assembled via IDBA-UD (V.1.1.3) [35] and SPAdes assembly software (v.2.0.5) [36] to generate contig sequences. The contigs were aligned with the virus database using BLAST software MEGA X ((V.10.0.5) to screen the target sequences. Based on the alignment results, parametric assembly was performed using BWA and SAMtools software (v1.8) [37] to obtain the optimized final viral contig sequence [38].

### 2.9. Phylogenetic Analysis

The *rotavirus* reference and gene sequences were downloaded from the NCBI website (accessed on 22 May 2025, from https://www.ncbi.nlm.nih.gov/). Multiple sequence alignments were performed using the ClustalW tool in MEGA X ((V.10.0.5, accessed on 22 May 2025, from https://www.megasoftware.net/) [39]. Nucleotide similarity was calculated from the p-distance in MEGA X, and phylogenetic trees were constructed using the proximity method based on the Kimura-2 parametric model with 1000 bootstrap replicates to assess the reliability of the clades [40].

## 3. Results

### 3.1. Quality Control Analysis of Viral Metagenomic Data

Metagenomic sequencing was performed on samples A1 through A5 using the Illumina NovaSeq 6000 sequencing platform. The datasets generated in the present study were submitted to GenBank under Bioproject ID PRJNA1215313 and PRJNA1226284 with NCBI SRA accession ID SRR32111960, SRR32419447, SRR32474053, SRR32485792, and SRR32501446. After quality control and host decontamination, the final number of valid reads for the five sets of samples ranged from 34,308,390 to 45,535,848. The number of assembled contigs ranged from 42,393 to 84,558. The Q20 values were all greater than 98%, the Q30 values were greater than 95%, the GC content ranged from 37% to 48%, and the single-base sequencing error rates were all lower than 0.03%, indicating good genome sequencing results and the high integrity and quality of the gene assembly (Table 1).

### 3.2. Diversity Analysis of Enteric RNA Viruses in Fawns

**Figure 1:**
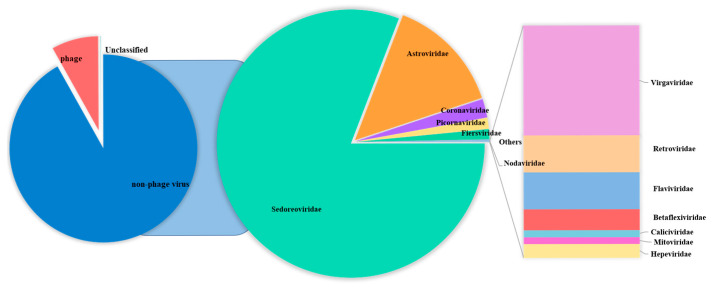
The pie chart shows the composition of fecal virus groups of diarrhea sika deer in different virus families.

**Figure 2:**
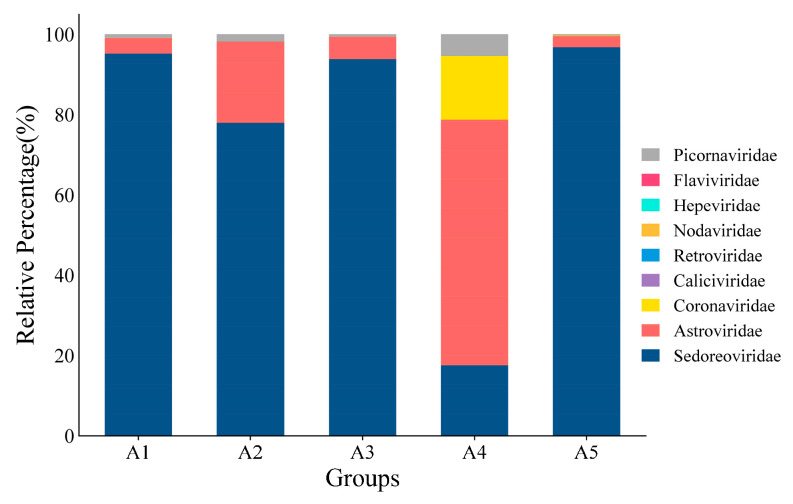
Statistical map of relative abundance at the family level.

The Blastn comparison analysis of nucleotide sequences indicates that a total of 291,008 assembled contigs exhibited a high degree of similarity with viral nucleotide sequences. The tentative viruses in the feces viral communities were distributed in 21 families [41,42,43,44] in 14 orders. A notable observation was the predominance of *Sedoreoviridae* and the family *Astroviridae* in the samples examined (Supplementary Tables S2 and S3), and the number of viral reads from each family was greater than 50. The distribution of mammalian viral reads in each library was analyzed according to the abundance of sequence reads (Figure 1). A large number of phages, which mainly belonged to the family *Siphoviridae*, were identified. In addition to bacteriophages, nine viral families are associated with diarrhea, including *Picornaviridae* [45], *Coronaviridae*, *Retroviridae*, *Hepeviridae* [46], *Flaviviridae*, *Nodaviridae* [47], *Caliciviridae* [48], *Astroviridae* [49], and *Sedoreoviridae* [50,51,52,53,54]. Among them, *Sedoreoviridae*, *Astroviridae*, *Coronaviridae*, and *Picornaviridae* ranked in the top 4 in terms of abundance at 82.10%, 14.25%, 2.28%, and 1.353%, respectively, as shown in Figure 2 and Table 2.

**Figure 3:**
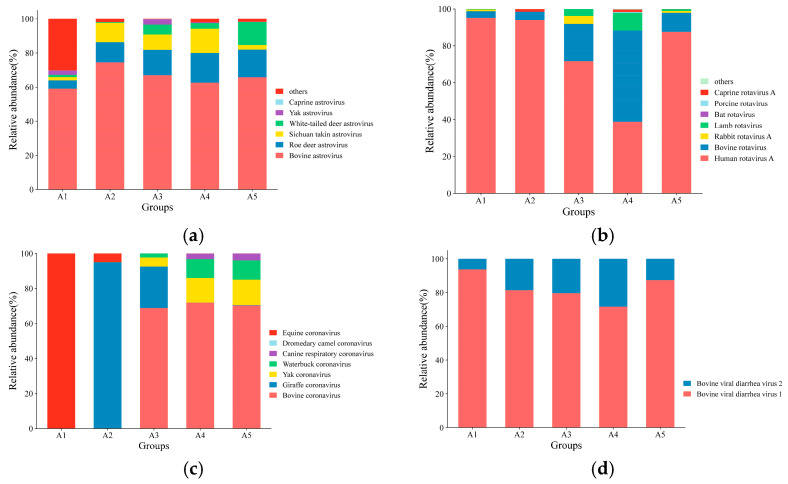
Relative abundance of viral communities in each group: (**a**) distribution of the Astroviri dae family; (**b**) distribution of the rotavirus genus; (**c**) distribution of the Coronaviridae family; (**d**) distribution of the Pestivirus genus.

The abundance of different viruses varied among the groups, and the distribution trends of the predominant viruses remained broadly consistent. Among the *Astroviridae* family, bovine astrovirus (BoAstV) had an abundance of more than 50% in all five groups, averaging 65.79%. The abundance of roe deer astrovirus was second highest, with an average of 13.02%, followed by Sichuan Takin astrovirus, with an average of 7.82%. Among the *rotavirus* genus, human rotavirus was the most abundant, with an average of 75.41%, whereas BRV was 17.67% on average. Among the *Coronaviridae* family, BCoV was the most dominant virus, averaging 42.22%, whereas giraffe coronaviruses and equine coronaviruses occupied 23.81% and 20.99%, respectively. BVDV type 1 was the dominant strain among the *Pestivirus* genus, with an average value of 82.79% (Figure 3).

### 3.3. Epidemiological Investigation of Enteric Pathogens in Fawns

BCoV, BRV, and BVDV were detected in 360 diarrhea samples using a qPCR detection kit (TopGeneBio, Qingdao, China). The positive rates of BCoV, BRV, and BVDV were 59.44%, 58.89%, and 21.67%, respectively (Table 3). This study revealed the occurrence of mixed infections with BRV and BCoV; BRV and BVDV; BCoV and BVDV; and BCoV, BRV, and BVDV. The mixed infection rates were 26.94%, 1.39%, 3.61%, and 16.11%, respectively (Table 3).

### 3.4. In Vitro Identification of Rotavirus

**Figure 4:**
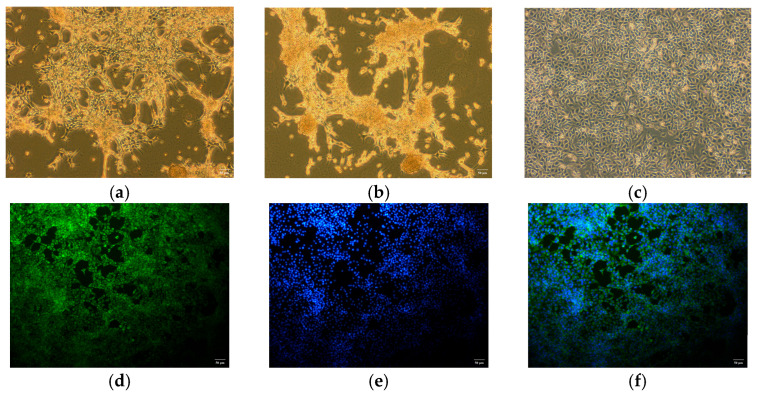
Isolation, cultivation, and immunofluorescence identification of rotavirus: (**a**) CPE of fourth-passage Vero cells infected with the SY1 isolate for 36 h. (**b**) CPE of fifth-passage Vero cells infected with the SY1 isolates for 24 h. (**c**) Normal Vero cells. (**d**) SY1-infected Vero cells stained with the RVA antibody (green). (**e**) The stained nuclei (blue) of Vero cells. (**f**) Merged image of Vero cells.

**Figure 5:**
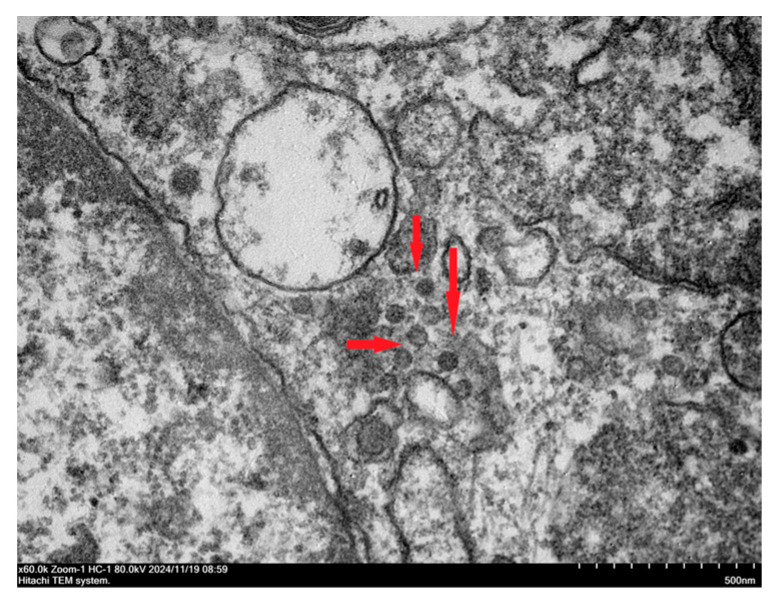
Electron microscopy image of RVA/Sika deer-wt/CHN/SY1/2024 virus particles (25,000×).

BRV-positive samples were inoculated into Vero cells for subculture. The results indicated that the cytopathic effect developed in the fourth subculture generation. The cells became rounder, the edges narrowed, and the volume decreased. Some cells shed and join in the cytoplasm, and a reticular network developed (Figure 4a). At the fifth passage, Vero cells inoculated with the pathogen demonstrated a cytopathic effect (CPE) 24 h after inoculation, with the CPE reaching 80% by 48 h (Figure 4b). Uninfected Vero cells maintained a clear morphology of irregular polygonal shapes and showed a gradual increase in cell density over time without any abnormal changes (Figure 4c). An immunofluorescence assay was performed with a monoclonal antibody against the VP6 protein of rotavirus as the primary antibody and FITC-conjugated goat anti-mouse IgG as the secondary antibody to confirm the rotavirus infection on Vero cells further. The results revealed that green fluorescence was present in Vero cells inoculated with the isolated strains (Figure 4d–f). At the same time, pure virus samples were prepared for electron microscopy examination. The results revealed that the virus particles were distributed in the cytoplasm of the diseased cells. They were spherical, similar to a wheel of virus particles, with an apparent bilayer capsid structure and a diameter between 65 nm and 70 nm (Figure 5). The strain isolated in this study was ultimately determined to be rotavirus and named RVA/Sika deer-wt/CHN/SY1/2024, abbreviated as SY1.

### 3.5. Genetype Analysis of Rotavirus

NGS technology was used to sequence the whole genomes of SY1 isolates, and 2,240,231 valid reads were produced. According to the latest classification and naming system established by the Matthijnssens, the nucleotide identity cutoff values for 11 gene segments of *rotavirus* Group A were as follows: 80% (G), 80% (P), 85% (I), 83% (R), 84% (C), 81% (M), 79% (A), 85% (N), 85% (T), 85% (E), and 91% (H). The genotype of SY1 was identified as G6-P[1]-I2-R2-C2-M2-A3-N2-T6-E2-H3 [55]. A BLAST search of the fragments generated from various isolates revealed high identity, and they were most closely related to sequences derived from cattle and humans. Among them, the nucleotide sequence identity of the VP1 gene fragment and the human rotavirus RVA/human-WT/HUN/BP1062/2004 strain was 99.76%; the nucleotide sequence identity of the VP4 gene fragment and the RVA/Cow-tc/CHN/SDA2 strain was 99.83%; the nucleotide sequence identity of the VP6 gene and the RVA/Bovine/HM26 strain was 96.6%; the nucleotide sequence identity of the VP7 gene fragment and the RVA/Yak-tc/China/F8 strain was 96.6%; the nucleotide sequence identity of the NSP1 gene fragment and the RVAYak-tc/CHN/HY-1 strain was 100.0%; and the nucleotide sequence identity of the NSP4 gene fragment and the RVA/Bovine/HM26 strain was 98.40% (Table 4).

### 3.6. Genetic Evolutionary Analysis

**Figure 6:**
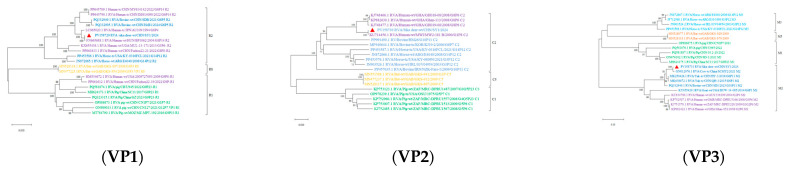

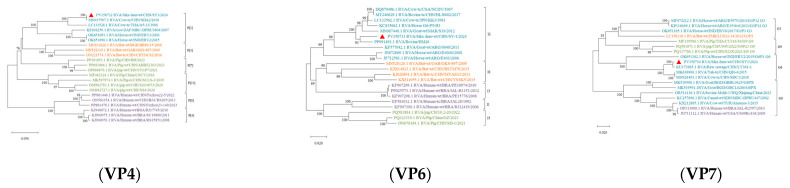
Phylogenetic tree of the nucleotide sequences of the VP1, VP2, VP3, VP4, VP6, and VP7 genes of RVA strains. Phylogenetic trees were constructed based on the nucleotide sequences of different gene segments of SY1 and other reference strains using a proximity analysis with 1000 bootstrap repeats. The RVA strains are color-coded: human strains are shown in purple; pig breed strains are shown in green; herbivore strains are shown in green; and bat strains are shown in orange. Red triangles highlight the RVA strain SY1 identified in this study. The scale representing evolutionary distance represents the nucleotide replacement rate at each site. The bootstrap value is displayed in the branch to the left of the main node above.

**Figure 7:**
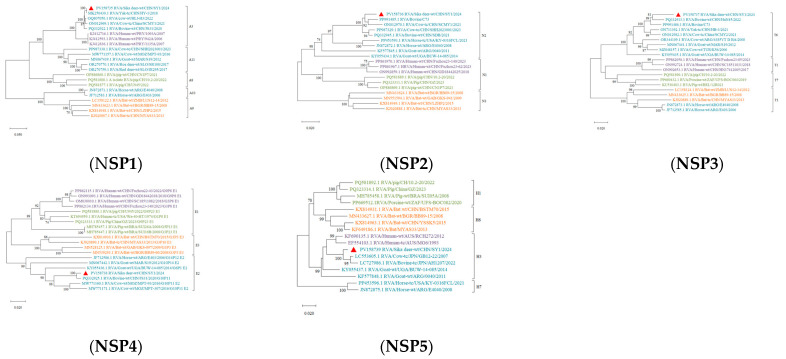
Phylogenetic tree of the nucleotide sequences of the NSP1, NSP2, NSP3, NSP4, and NSP5 genes of RVA strains. Phylogenetic trees were constructed based on the nucleotide sequences of different gene segments of SY1 and other reference strains using a proximity analysis with 1000 bootstrap repeats. The RVA strains are color-coded: human strains are shown in purple; pig breed strains are shown in green; herbivore strains are shown in green; and bat strains are shown in orange. Red triangles highlight the RVA strain SY1 identified in this study. The scale representing evolutionary distance represents the nucleotide replacement rate at each site. The bootstrap value is displayed in the branch to the left of the main node above.

Reference sequences were downloaded from the NCBI database based on whole genome sequencing results. MEGA X software (V.10.0.5) was used to analyze the genetic evolution of all the genes encoding structural and nonstructural proteins. All sequences of information on Phylogenetic trees are shown in Supplementary Table S4. The results revealed noticeable genetic differences between the rotavirus (RVA) strains isolated from sika deer, pig, bat, and human strains. Most of the 11 gene segments of this RNA strain clustered significantly more closely with bovine RVA strains than with porcine, mouse, or human RVB strains, suggesting that sika deer rotavirus may have originated in cattle (Figure 6 and Figure 7). In addition, the phylogenetic analysis revealed that the VP6 genes of the SY1 isolate clustered in the same evolutionary branch as the bovine RVA genes, and the VP3, VP4, and NSP1 genes clustered in the same evolutionary branch as the cow RVA genes. The VP1, VP2, and NSP5 genes clustered in the same evolutionary branch as the human rotavirus genes. This pattern of gene grouping across species indicates gene rearrangement in the sika deer rotavirus.

## 4. Discussion

Diarrhea significantly impacts sika deer production [21,56]. Diarrhea severely hinders the prevention and control of animal diseases, which not only affects the health of sika deer but also causes significant economic loss. The pathogens causing diarrhea in sika deer are not completely clear. The known RNA viral pathogens are BVDV [20], BRV [57], and BCoV [58], but many remain unidentified in sika deer. Therefore, more accurate data on the epidemiology and etiology of the causative pathogens of diarrhea in sika deer are essential for developing integrated preventive measures.

Next-generation sequencing (NGS), which has a short cycle time and high-throughput characteristics, can simultaneously obtain millions of short sequence reads. The development of this technology has not only reduced sequencing costs and improved sequencing efficiency but also made it possible to analyze the transcriptome and genome of a species in detail [59,60]. This technology is also widely used in many research fields, including marine ecology, agriculture, human genetics, and diagnostics. In marine ecology, viral metagenomics has contributed to understanding marine biodiversity and ecological traits and provided a solid scientific foundation for safeguarding marine ecosystems [25]. In agriculture, this technology helps to monitor and control viral diseases that affect crop growth, thereby improving agricultural production efficiency and crop yield [61]. In animal husbandry, this technology can significantly improve the detection of viral diseases, thereby reducing the number of animal deaths [14]. In human genetic research, viral metagenomics provides a new approach to the early diagnosis and treatment of diseases [29].

This study used viral metagenomic sequencing to examine RNA viruses in sika deer diarrhea samples. Considering the high sensitivity of the NGS technology, a total of 360 samples were collected carefully and directly from the anus of fawns with diarrhea to avoid contamination by factors from the environment. All samples were pooled into 12 groups and then used for RNA isolation. However, only five groups of RNA samples met the sequencing requirements after quality control and were subjected to sequencing in this study. However, with a limited sample size, viral metagenome sequencing revealed that the viral reads from the fecal samples of the fawns were mainly from 21 viral families in 14 viral orders. We have discovered the viral genomes including the *Picornaviridae*, *Coronaviridae*, *Retroviridae*, *Hepeviridae*, *Flaviviridae*, *Nodaviridae*, *Calici-viridae*, *Astroviridae*, and *Sedoreoviridae* families. The major viruses in high abundance were astrovirus, rotavirus, coronavirus, and viral diarrhea virus. In addition, we also identified a large number of phage viruses in the feces of diarrheic deer. Our research has enriched our knowledge of the diversity of virus communities in diarrheal sika deer. In previous studies, numerous bovine-related viruses, such as BRV, BCoV, and BVDV, have been detected in sika deer diarrhea samples, and these viruses account for a reasonably significant proportion of the viral metagenome. This study confirmed that sika deer are a potential reservoir for these viruses. This finding indicates that deer farms’ disease prevention and control procedures should receive more attention.

*Rotavirus* is an important pathogen that can cause acute viral gastroenteritis and diarrhea in humans, domestic animals, and wild animals [62,63]. Several instances of rotavirus infection have been identified in Cervidae from other countries, but few from China. In this study, we discovered that the prevalence of rotavirus was 58.89% using viral metagenomic detection technology. It is the primary RNA virus associated with diarrhea in deer. This result suggested that the harm caused by rotavirus to sika deer should be considered. RVA belongs to the Reoviridae family and has a segmented double-stranded RNA (dsRNA) genome of 11 distinct linear segments. Among these genes, VP6 is generally conserved in various rotavirus strains and may be classified into nine categories based on the gene sequence: A, B, C, D, F, G, H, I, and J [64]. Among these nine rotaviruses, rotavirus A (RVA) mainly infects humans, and G1P[8], G2P[4], G3P[8], G4P[8], G9P[8], and G12P[8] have been shown to dominate global rotavirus transmission in humans [65]. Additionally, each rotavirus gene segment contains encoding genes. The unusual structure of rotavirus allows it to reassign different types of viruses during reproduction, resulting in new antigenic variations. A previous study by Li et al. [66] discovered a pig strain of rotavirus, which contained five gene fragments, namely, VP1, VP2, VP3, NSP3, and NSP4, highly similar to sequences from the herbivore rotavirus. In this study, the whole-genome sequencing analysis revealed that the rotavirus isolated from sika deer feces was highly similar to human rotavirus, whereas the remaining fragments were highly similar to sequences from bovine rotavirus. These findings further confirmed the complexity of rotavirus transmission and evolution among different hosts and their diversity and dynamic changes at the genetic level.

Since its first detection in the United States in 1972, BCoV has been recorded worldwide [67,68]. The virus has been reported throughout Europe, America, Asia, and Africa [69]. The virus spreads mainly through the digestive and respiratory tracts, with ill or healthy animals serving as the primary source of infection. BCoV may infect numerous domestic and wild ruminants, including buffalo, sheep, goats, dromedary camels, llamas, alpaca, deer, bison, antelope, giraffe, and ibex, especially cattle [70]. BCoV has also been detected in dogs and humans [71,72]. Yokoi et al. [73] tested 179 domesticated sika deer in Japan for BCoV and reported a positive rate of 1.1%. In 2022, Liu et al. [74] reported no BCoV infection in a study of diarrheal infections in sika deer in Shaanxi, China. The broad spread of BCoV threatens cattle health and may pose potential risks to the health of other animals and even humans. In the present study, the prevalence of BCoV in sika deer fawns was as high as 59.44%. Therefore, strengthening the monitoring and research of BCoV is highly important for preventing and controlling the spread of the virus and promoting the development of the animal husbandry industry.

BVDV, a member of the *Pestivirus* genus in the Flaviviridae family, is an enveloped, single-stranded, positive-sense RNA virus [75]. Infected cattle, particularly juvenile animals, can experience diarrhea, high fever, leukopenia, mucosal erosion, and exfoliation. Infection with this virus can also result in abortion, stillbirth, mummified or malformed fetuses, and reproductive dysfunction in pregnant animals [76,77]. The virus has a wide host range. It can infect pigs, sheep, buffalo, camels, sika deer, and other animals [78,79,80]. Several investigations have documented the occurrence of BVDV infection in cervids. Antonio et al. conducted a serological examination of BVDV in white-tailed deer in northern Mexico, and the results revealed that the rate of BVDV antibody detection in white-tailed deer was 63.5% [81]. In China, the BVDV infection rate in older deer can reach 34.1%, whereas the BVDV infection rate in fawns ranges from 60.0% to 86.7% [20]. Our study tested 360 fecal samples from sika deer fawns and discovered that the BVDV infection rate was 21.67%. The low infection rate might be attributed to the sample collection region, sample number, detection technique sensitivity and specificity, and virus epidemic dynamics. In addition, BVDV may infect sika deer through cross-species transmission between cattle and other domestic animals, causing clinical symptoms such as diarrhea, emaciation, and even death, posing a threat to the sika deer breeding industry. Therefore, although BVDV mainly infects cattle, its potential impact on Cervidae cannot be ignored, and prevention and control measures need to be strengthened in breeding practices to protect the health of deer populations.

In conclusion, this study used viral metagenomics to analyze diarrhea-associated RNA virus infection in sika deer fawns in Jilin Province. The results showed that the viral pathogens were mainly from 21 viral families in 14 viral orders. Many of the novel viruses remain for further study. However, the present study revealed that Chinese sika deer were mainly infected with BCoV, BRV, and BVDV, which has a certain warning effect and provides data supporting the prevention, control, and diagnosis of diarrhea in sika deer. The pathogens associated with fawn diarrhea will be investigated in future studies. This can aid in our comprehension of the genetic traits, infection mechanisms, and biological behavior of these viruses, as well as aid in the prediction of potential intra- or interspecies transmission of these viruses. It can also serve as a foundation for future research into the detection, treatment, and prevention of emerging viral infections in mammalian and human hosts.

## Figures and Tables

**Table 1:** Sample sequencing information.

Groups	Clean Reads	All Tran Num	Clean Bases	Error Rate	Q20	Q30	GC Content
A1	37,291,842	56,013	5.59 G	0.02	98.56	95.57	40
A2	45,535,848	84,558	6.83 G	0.02	98.63	95.75	44.53
A4	36,317,604	53,317	5.45 G	0.02	98.47	95.34	47.47
A3	34,308,390	54,727	5.15 G	0.02	98.43	95.22	39.22
A5	40,418,916	42,393	6.06 G	0.02	98.42	95.15	37.46

**Table 2:** List of relative abundance at the family level.

Family/Groups	A1	A2	A3	A4	A5	Total
*Sedoreoviridae*	95.21%	78.00%	93.85%	17.55%	96.82%	82.10%
*Astroviridae*	3.932%	20.27%	5.582%	61.17%	2.779%	14.25%
*Coronaviridae*	0.007009%	0.01029%	0.004904%	15.90%	0.1652%	2.279%
*Caliciviridae*	0	0	0.0007864%	0.009401%	0.00004804%	0.001481%
*Retroviridae*	0.003899%	0.01039%	0.006507%	0.02781%	0.001106%	0.007843%
*Nodaviridae*	0.001326%	0	0	0	0	0.0003038%
*Hepeviridae*	0.001023%	0.004980%	0.0006945%	0.0004869%	0.0003797%	0.001258%
*Flaviviridae*	0.0003907%	0.01831%	0.006307%	0.01925%	0.003013%	0.007497%
*Picornaviridae*	0.8440%	1.690%	0.5484%	5.318%	0.2328%	1.353%

**Table 3:** Prevalence of the main pathogens causing diarrhea in Sika deer.

Pathogens	Positive Samples	Negative Samples	Positive Rate/%
BRV	212	148	58.89
BCoV	214	146	59.44
BVDV	78	282	21.67
BRV and BCoV	97	263	26.94
BRV and BVDV	5	355	1.39
BCoV and BVDV	13	347	3.61
BRV, BCoV and BVDV	58	302	16.11

**Table 4:** Information on the 11 gene segments of the SY1 strain.

Gene	Length (bp)	Coding Region (bp)	Genotype	Virus with the Highest Identity	Identity (%)	GenBank ID
VP1	3302	3267	R2	Human-wt/HUN/BP1062	99.76	PV158729
VP2	2690	2260	C2	Human-wt/MWI/MW2-181_B	100	PV158730
VP3	2508	2508	M2	Cow-tc/China/SCMY2	99.48	PV158731
VP4	2362	2088	P[1]	Cow-tc/CHN/SDA2/G6P[1]	99.83	PV158732
VP6	1352	1215	I2	RV/Bovine/HM26	96.60	PV158733
VP7	1062	981	G6	Yak-tc/China/F8	96.81	PV158734
NSP1	1579	1476	A3	Yak-tc/CHN/HY-1	100.00	PV158735
NSP2	1055	996	N2	Bovine/C73	97.43	PV158736
NSP3	1074	966	T6	Lamb/CHN/LLR	98.24	PV158737
NSP4	749	567	E2	Bovine/HM26	98.40	PV158738
NSP5	597	597	H3	Cow-tc/JPN/GB12-22	98.32	PV158739

## Data Availability

The genomic sequences obtained in this study have been deposited in the NCBI GenBank repository under the accession numbers provided directly within the manuscript.

## Supplementary Materials

The following supporting information can be downloaded at: https://www.mdpi.com/article/10.3390/v17060803/s1. Table S1: Criteria for genetic and non-genetic characterization of ICTV-presumed new virus genera or species. Table S2: Viral expression abundance at different levels of classification. Table S3: Information of virus sequence information in this study. Table S4: Reference sequences used for the construction of phylogenitical trees.
